# The Relation of Contract Surgeons of the Several Railroads, to the General Profession

**Published:** 1891-07

**Authors:** 


"THE RELATION OF CONTRACT SURGEONS of the different railroad systems, to the general profession," is the title of a memorial paper sent up by the West Virginia State Medical Association and read at the Washington meeting of the American Medical Association. It was, on motion, referred to a committee of one member from each State Medical Association in affiliation; and Dr. D. R. Wallace, of Waco, was appointed to represent Texas. No chairman was appointed, but Dr. Jerome Cochran, of Alabama, being named first on the committee, assuming the daties of chairman, *pro tem.*, has issued a circular letter—calling on the members of the committee to communicate with Dr. W. B. Atkinson, general secretary of the A. M. A., indicating their choice for chairman. After organization the committee will, we suppose, hold a conference and report to the A. M. A. their construction of Section 6, Article 5 of the code, (the section specifically referred to in the memorial "Of the duties of physicians to each other"), giving their opinion of—or, defining in more exact terms—the much discussed status of contract surgeons.

This memorial is published in full in the Association's journal of May 16, page 715. Those desiring further information on the subject are referred to it.

The JOURNAL is rejoiced to see the question taking shape to be brought before the only tribunal having jurisdiction in the premises and hopes to see it satisfactorily settled. It has been much discussed—and has given rise to much dissention and even bitterness, amongst the "ins" and "outs", some of the latter claiming that it is unethical to hold such relation to a railroad corporation.

All old members of the Association (T. S. M. A.) will recall with a smile, the indignation, not to say *wrath* of a certain super-ethical(?) "old cove" "(a member) who attended the Waco meeting in '81, it was alleged, for the purpose of purging the Association of all such trash" (?) as railroaders, they being "too unethical" he said for *his* pure, (simon) virtuous, uncontaminated presence, or "bust the Association up"; and how dear old Ashbel Smith, then President, mildly sat down upon him, gently intimating that if he did didn't like the company he was at liberty to get out. These "too-utterly unethical" fellows are still "in it," these railroaders, we mean; and the old gent is still manœuvering, and unhappy because they are so "unethical."

We shall look anxiously for the definitions; it is a vexed question; one the JOURNAL has often been requested to discuss, but has forborne. That the contract system is abused, there is no doubt; but the changed times and circumstances render it necessary that medical men should be connected with and representative of the interests of these large corporations in some way—and the one in vogue seems to be as unobjectionable as any yet devised. Much can be said on both sides, but in anticipation of the approaching conference we have nothing to say.

